# Innovations in Thermal Processing: Hot-Melt Extrusion and KinetiSol® Dispersing

**DOI:** 10.1208/s12249-020-01854-2

**Published:** 2020-11-08

**Authors:** Deck Khong Tan, Daniel A. Davis, Dave A. Miller, Robert O. Williams, Ali Nokhodchi

**Affiliations:** 1 Pharmaceutics Research Laboratory, Arundel Building, School of Life Sciences, University of Sussex, Brighton, BN1 9QJ UK; 2 College of Pharmacy, The University of Texas at Austin, Austin, Texas 78712 USA; 3 DisperSol Technologies, LLC, 111 W. Cooperative Way, Building 3, Suite 300, Georgetown, Texas 78626 USA

**Keywords:** hot-melt extrusion, KinetiSol Dispersing, amorphous solid dispersion, pharmaceutical co-crystals, in-line process analytical technology

## Abstract

Thermal processing has gained much interest in the pharmaceutical industry, particularly for the enhancement of solubility, bioavailability, and dissolution of active pharmaceutical ingredients (APIs) with poor aqueous solubility. Formulation scientists have developed various techniques which may include physical and chemical modifications to achieve solubility enhancement. One of the most commonly used methods for solubility enhancement is through the use of amorphous solid dispersions (ASDs). Examples of commercialized ASDs include Kaletra®, Kalydeco®, and Onmel®. Various technologies produce ASDs; some of the approaches, such as spray-drying, solvent evaporation, and lyophilization, involve the use of solvents, whereas thermal approaches often do not require solvents. Processes that do not require solvents are usually preferred, as some solvents may induce toxicity due to residual solvents and are often considered to be damaging to the environment. The purpose of this review is to provide an update on recent innovations reported for using hot-melt extrusion and KinetiSol® Dispersing technologies to formulate poorly water-soluble APIs in amorphous solid dispersions. We will address development challenges for poorly water-soluble APIs and how these two processes meet these challenges.

## INTRODUCTION

**Fig. 1:**
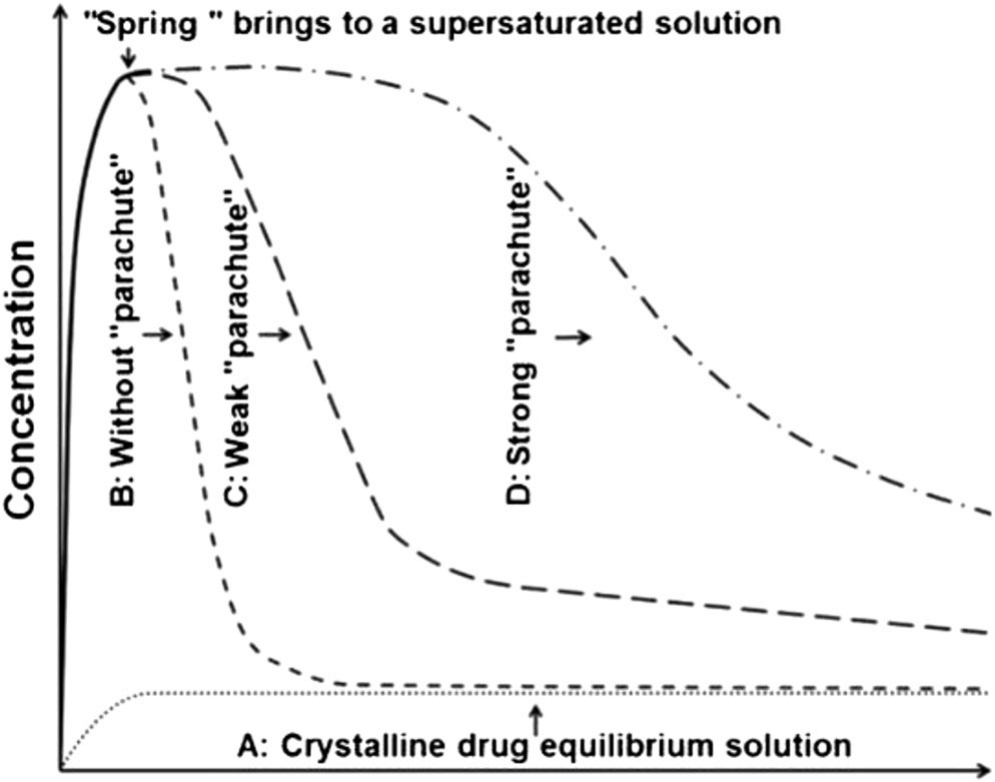
Schematic diagram of the Spring-parachute model approach for solubility enhancement (adapted with permission from (5))

It is estimated that about 40% of the drugs currently on the market and up to 70–90% of the drugs in the pharmaceutical discovery pipeline are poorly water-soluble (1–3). The majority of these poorly water-soluble drugs in the discovery pipeline are weak acids or weak bases, which are further classified as biopharmaceutical classification system (BCS) class-II and class IV drugs depending on their permeability (3,4). Therefore, improving solubility and dissolution is essential to enhance drug efficacy and delivery. A theoretical model, the ‘spring-parachute model,’ is often used to illustrate different scenarios of solubility enhancement (depicted in Fig. 1). Specifically, the spring-parachute model proposes that the drug is rapidly released into solution, in a supersaturated state, that is above its equilibrium solubility and then maintained in this state for as long as possible (6–8). The most common approaches proposed to meet this model include the crystalline approach (5,9,10), non-polymeric approach (11–13), and amorphous solid dispersion (ASD) (14,15).

Several technologies alter the apparent solubility and dissolution properties of active pharmaceutical ingredients (API) to achieve the aim of prolonged solubility enhancement. Some processes are solvent-based, while others are solvent-free. The solvent-based processes include solvent evaporation and spray drying. Whereas, the solvent-free processes are often achieved through thermal processes; an example of the solvent-free thermal process is hot-melt extrusion (HME) and KinetiSol® Dispersing (KSD). HME is a prevalent technology in the plastics industry that was developed over a century ago and only recently adapted to be used by the pharmaceutical industry (16). The increased interest in HME can be attributed to its versatility and robustness of the technology in which it can be easily scaled according to the specific application requirement, and it can be incorporated with other novel systems for extended pharmaceutical applications. However, this technology has been modified from the ones being used in the plastics industry to meet the regulatory requirements and good manufacturing practices for pharmaceutical applications and manufacturing (17). Similar to HME, KSD, which is a high-energy mixing process that does not use externally applied heat, originated from the plastics and recycling industries and has been modified accordingly for pharmaceutical applications (18). The focus of this article will be on reviewing, HME and KSD technologies, the two major solvent-free thermal processes in the pharmaceutical industry for solubility enhancement through formation of an ASD.

A solid dispersion is defined as a dispersion of one or more active ingredients that are dispersed or dissolved in an inert carrier/polymeric matrix in the solid-state, which can be prepared by melting/fusion method and solvent method (19). Solid dispersions aim to reduce the particle size of the API to improve the solubility and dissolution rate. In pharmaceutical applications, amorphous solids offer the advantages of higher solubility, higher dissolution rate, and better compression characteristics as compared to the corresponding crystalline solid (20). This benefit is a result of the disruption of the crystalline lattice, which creates a meta-stable state decreasing the activation energy, ultimately increasing apparent solubility (21). Amorphous solid dispersions (ASDs) are used to improve the solubility and bioavailability of poorly water-soluble APIs, as highlighted by FDA approved ASDs being either BCS Class II or IV drugs. Apart from that, ASDs benefit from their ability to be incorporated into solid dosage forms, which is still the preferred route of drug administration (22). Oral dosage forms are preferred due to the ease of administration, better handling convenience, and patient compliance, as well as relative product stability (23).

Amorphous solids are metastable and can recrystallize when there is sufficient molecular mobility in the mixture (24). However, the polymer in the ASD plays an important role in stabilizing the API in the amorphous state by API-polymer interactions (25). Therefore, the selection of suitable polymers is essential to inhibit the crystallization process. The polymer needs to play a role in limiting the drug molecule’s mobility, lowering the nucleation and crystallization process, increasing the activation energy of nucleation, and increasing the glass transition temperature of the mixture to inhibit the recrystallization of the drug. Some of the most commonly used polymers for ASD production in HME are polyethylene glycol (PEG), polyethylene oxide (PEO), polyvinyl alcohol (PVA), crospovidone, polyvinylpyrrolidone (PVP), hydroxypropyl methylcellulose (HPMC), hydroxypropyl methylcellulose acetate succinate (HPMC-AS), polyvinylpyrrolidone-vinyl acetate (copovidone).

Chemically and physically stable ASDs have been prepared using both the HME and KSD technologies. However, both technologies still pose challenges for pharmaceutical applications. Degradation of the materials can be caused during the HME and KSD processes by elevated temperatures and excessive shear exerted during the fusion process, thus requiring adjustments to be made to overcome degradation. As amorphous drugs exist in a higher energy state, they are also much more vulnerable to chemical degradation (26–28). In a study, HME was used to produce an albendazole ASD, and severe chemical degradation was observed due to the long residence time, high shearing forces, and elevated temperature caused during the extrusion process (15). However, thermal degradation can sometimes be avoided, depending on the specific API, by adjusting the processing conditions of the extruder, such as the barrel temperature and screw speed. Optimization of the processing conditions of HME can prevent degradation of materials, while at the same time ensuring complete amorphous conversion. However, optimizing processing conditions can only minimize degradation to a certain extent. This is because chemical degradation may occur from the incompatibilities of formulation excipients with API (*i.e.*, polymeric acetate groups promoting ester hydrolysis of the API) (29–31)**.** When minimizing thermal degradation, the optimization process can be achieved efficiently and effectively using in-line PAT, as discussed in the later section.

The chemical stability and solubility of ASDs are closely related to the thermal history of the materials being processed. The preparation of formulations at different temperatures may impart varying levels of molecular mobility, which will affect the crystallinity of the materials (32,33). In thermal processes, such as HME and KSD, the temperature of the materials drops rapidly upon exiting the equipment, immediately locking the drug particles in its position in the amorphous state. As these processes are fusion-based methods, the tendency of crystallization is generally lower than solvent-based methods such as spray drying (34,35). It was shown that the processing technologies do not affect the thermal characteristics such as the glass transition temperature of the materials (36). However, the processing techniques will affect the crystallization kinetics and the tendency for recrystallization of the prepared ASD (37). In most processes, the processing temperature is still a critical factor affecting the properties such as enthalpic relaxation, crystallization, and moisture absorption of the product (38).

The nucleation process and crystal growth contribute to the crystallization process, which can affect the physical stability of the ASD (39). Different processing methods exhibit different nucleation behavior. Generally, HME utilizes a high degree of mixing to provide sufficient energy for the intimate mixing of the API and polymer (40). This intimate mixing can have a positive effect on physical stability. The high shear rate of KSD accelerates the dissolution kinetics of the API in the polymeric carriers. The physical stability of the ASD depends on the converting power of the technology to change the material from crystalline to amorphous form. HME has high converting power, but it is highly dependent on the screw designs and configuration as well as other processing conditions (41). Another challenge for ASD is to avoid crystallization upon contact with the dissolution media. The polymer provides stabilization to the API in the supersaturated state by remaining in close contact with the API to prevent solvent-mediated crystallization. HME and KSD can effectively prepare ASDs with a high degree of dispersion of the crystalline API due to high shear mixing. This will also result in superior stability. Generally, APIs need to be homogeneously distributed in the polymeric carrier for enhanced drug release.

## HOT-MELT EXTRUSION

**Fig. 2:**
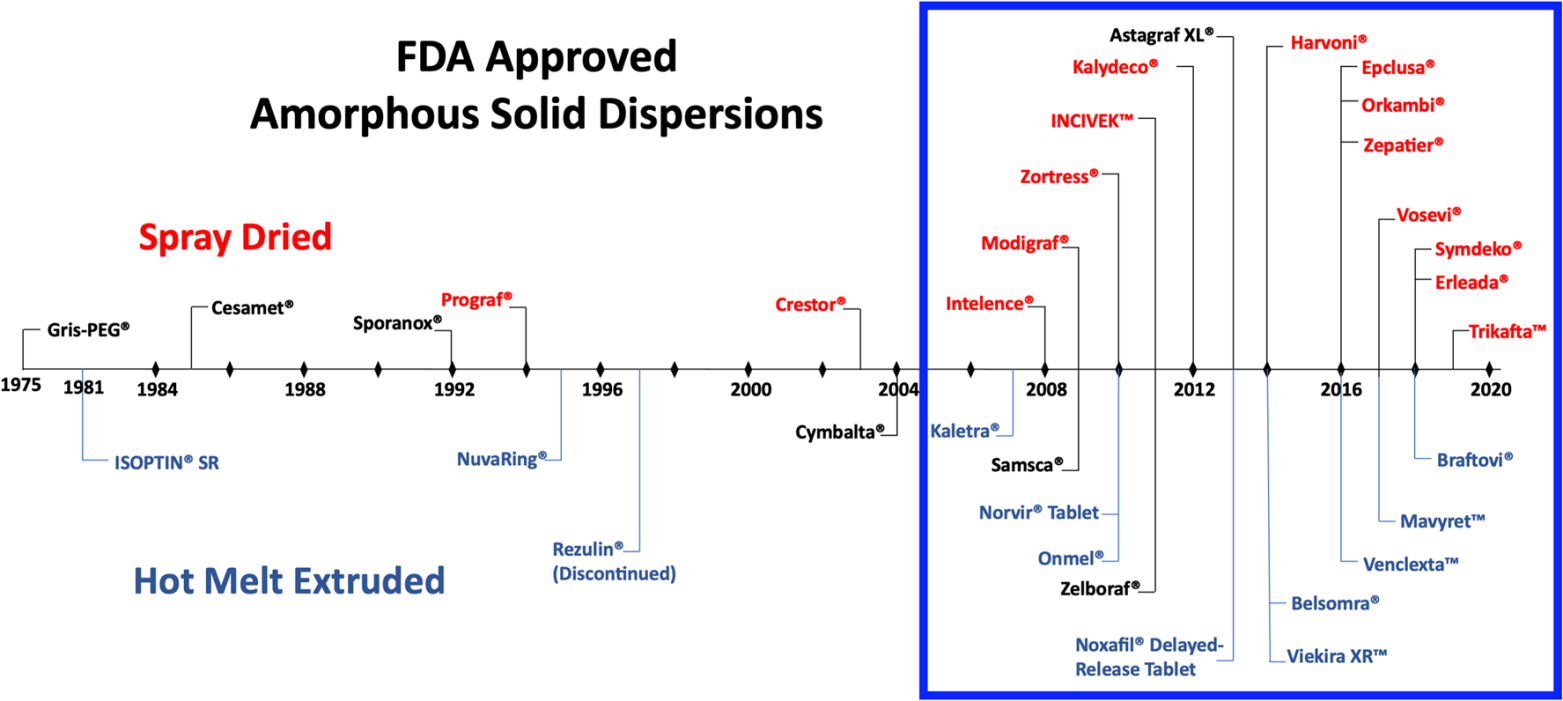
Timeline of FDA-approved amorphous solid dispersion products. The coloring scheme helps to differentiate the different processes used to create amorphous solid dispersions. Red indicates spray drying; blue indicates hot-melt extrusion, and black are other processing methods (adapted with permission from (44))

Hot-Melt Extrusion has been utilized since the 1930s in the food and plastics industries (42,43). In general, HME is a process of shearing and mixing different materials using screw elements at an elevated temperature to melt and intimately mix the drug-containing composition. HME is now one of the most commonly employed thermal processing techniques in the pharmaceutical industry (Fig. 2). This is attributed to the versatility of HME in addressing the requirement and demand of quality by design (QbD) and process analytical technology (PAT) as introduced by the US Food and Drug Administration (FDA) for quality control and assurance of pharmaceutical manufacturing. Traditional pharmaceutical manufacturing involves batch production, which contains batch-to-batch variations, requires large production volume and inconsistent quality. Batch manufacturing also has higher wastage of raw materials, long waiting, and throughput times (45). Therefore, regulatory bodies, such as the FDA, proposed continuous processing and manufacturing, thus discouraging batch production. This implementation can satisfy flexible market demands, reduce operational complexities and interruptions (46). In the last decade, a combination of increased pressure from regulating bodies and advances in our understanding of ASDs has integrated HME as an essential manufacturing process in the pharmaceutical industry (47). The pharmaceutical adaptation of ASDs began in 2005, with to-date 25 products being approved by various methods; before this point, only six ASDs were granted FDA approval (Fig. 2).

From 1997 to 2009, Rezulin®, Kaletra®, and Norvir® were the only three ASD produced by HME that received FDA approval (48,49). In a similar period, from 2009 to 2019, six ASD products manufactured by HME received FDA approval. Currently, the primary pharmaceutical applications for HME is to produce solid dispersions, taste masking of bitter APIs, and increase the solubility, dissolution, and overall bioavailability of poorly water-soluble APIs.

**Fig. 3:**
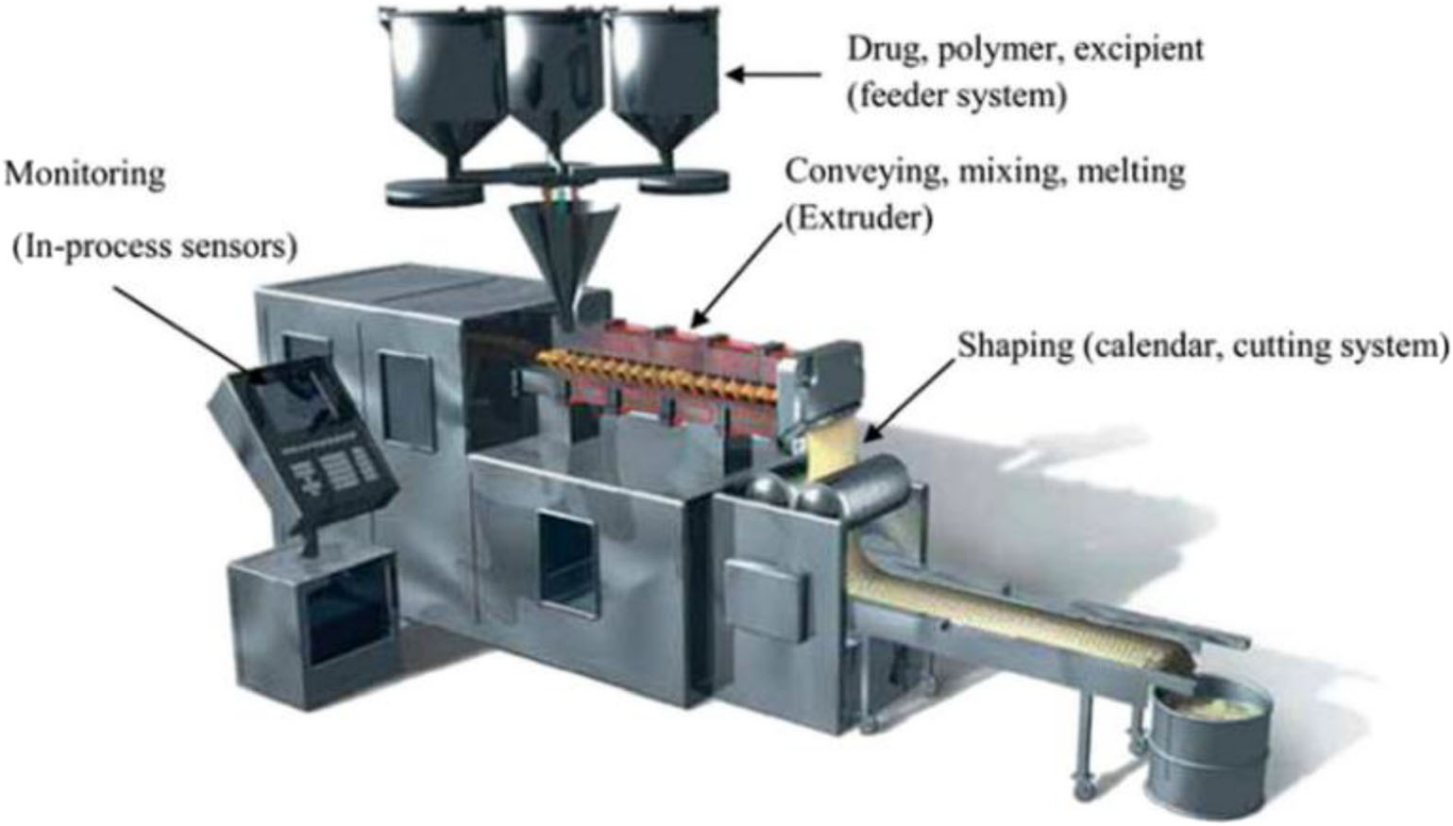
A typical pharmaceutical hot-melt extruder (adapted with permission from (50))

**Fig. 4:**
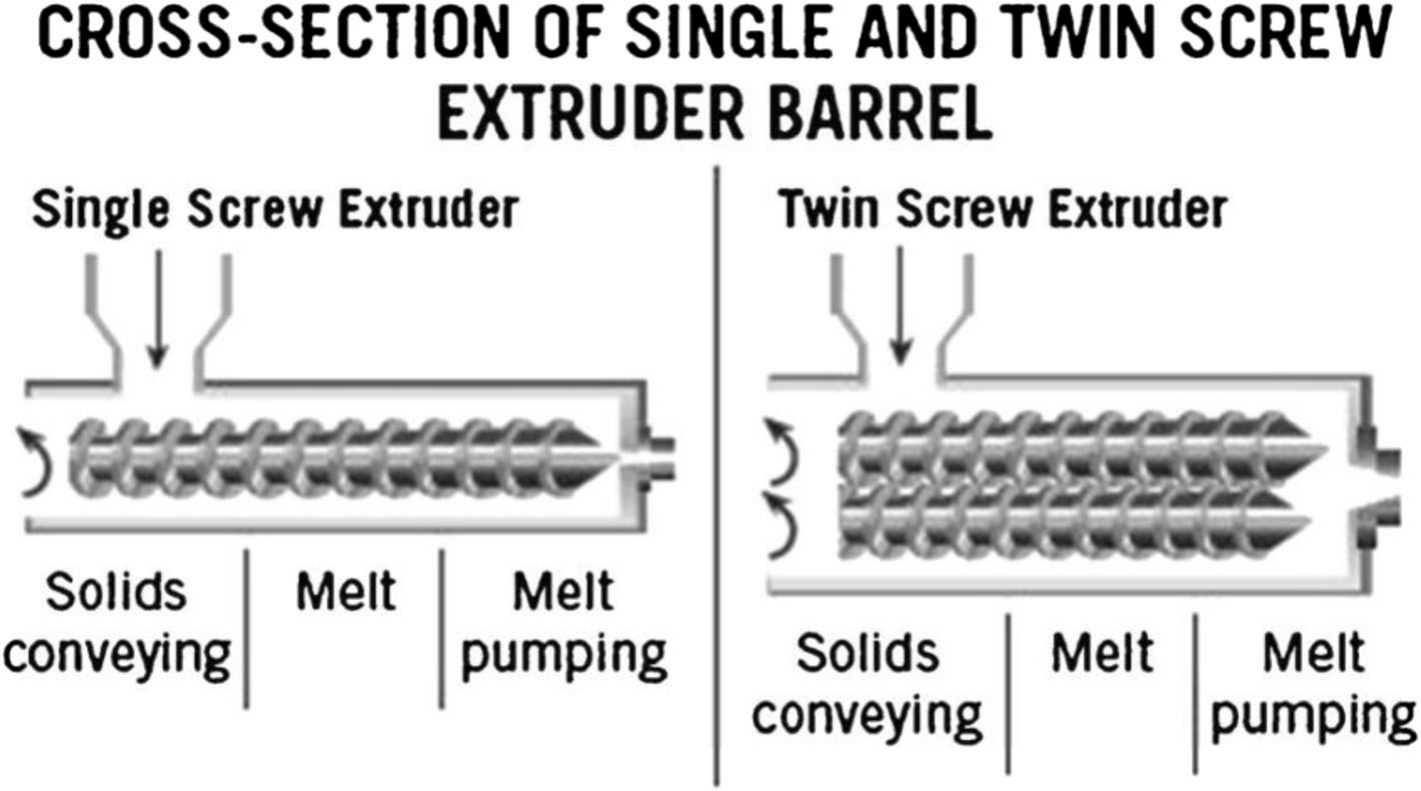
Cross-section of a single and twin-screw extruder (adapted with permission from reference (51))

**Fig. 5:**
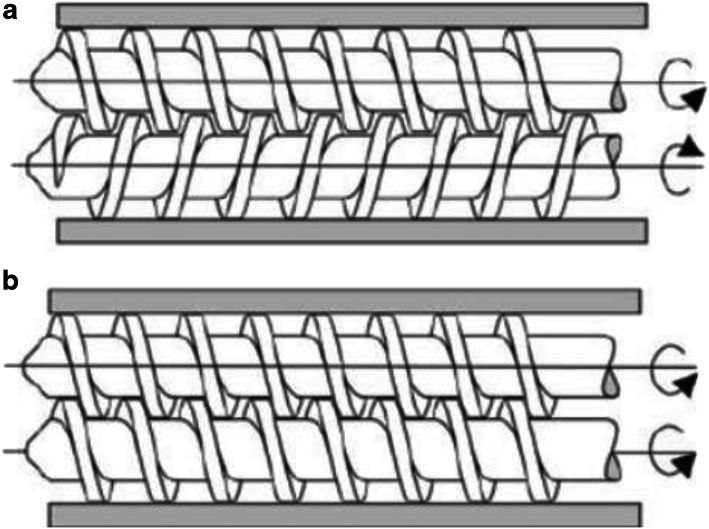
Twin-screw extruder barrel. **a** Counter-rotating screws. **b** Co-rotating screws (reproduced with permission from reference (54))

The HME process consists of several necessary components such as the feeder system, the barrel and screws for mixing and conveying of materials, heating elements for melting, the motor for controlling the screw speed, and a die for shaping the materials. A control system is incorporated in the extrusion process to control the processing conditions such as the feeding rate, rotating screw speed, processing temperature, *etc.* A diagram of a typical hot-melt extruder for pharmaceutical applications is depicted in Fig. 3. HME is generally classified into two categories: single-screw extruder and twin-screw extruder (Fig. 4). The single-screw extruder is commonly used to produce filaments (52) and films (28,29), whereas the twin-screw extruder has found utility in producing amorphous solid dispersions (53) (ASD) and pharmaceutical co-crystals. Twin-screw extruders can be further classified according to the direction of screw rotation: co-rotating or counter-rotating (Fig. 5). Twin-screw extrusion is generally preferred as the shearing provided by two rotating screws can ensure homogenous mixing. The high mixing efficiencies of the twin-screw extruder can ensure better repeatability and comply with the good-manufacturing practice for the manufacturing of pharmaceutical products (55).

## RECENT INNOVATION FORMULATIONS *VIA* HOT-MELT EXTRUSION

### Amorphous Solid Dispersions

HME, in particular twin-screw extrusion, is a robust processing method in producing ASDs. The rotating screws in twin-screw extrusion provide dispersive and distributive mixing of the API with polymeric excipients, providing enhanced mixing in comparison to single-screw extrusion (49). Twin-screw HME is also easily scalable, ensuring cost-effective large volume production. The ability to produce ASDs cost-effectively at a large volume is a significant advantage over other solvent-based ASD preparation techniques, such as spray drying. The elimination of the use of a solvent in HME also makes it a green and environmentally friendly process, which is much preferred (56). Sarabu et al. investigated the effect of different grades of hydroxypropyl methylcellulose acetate succinate (HPMC-AS), LG, MG, and TG, on the extrusion process, dissolution and stability of the ASDs produced using HME, where nifedipine (NFD) and efavirenz (EFZ) were used as model drugs (57). HPMC-AS is one of the most commonly used materials for ASD preparation, as it seems to exhibit an inhibitory effect on drug precipitation and crystallization while providing enteric release characteristics. However, its application in HME is limited due to the high processing temperatures of HME; the minimum processing temperature of pure HPMC-AS was 170°C, with degradation onset starting at 180°C. Despite the small extrusion window, the study here proved that it is possible to produce ASDs using HPMC-AS *via* HME when the API exhibits a plasticizing effect. The stability of the ASD is dependent on the physicochemical properties of the API and polymeric excipient, as well as the interactions between the API and polymer. The results showed that the combination of MG grade HPMC-AS with NFD and LG grade HPMC-AS with EFZ achieved the most significant “spring and parachute” effects for prolonged durations. The ASDs were stable up to 3 months when being tested at an environment of 40°C and 75% relative humidity (RH). Kallakunta et al. produced stable ASDs with a low glass transition temperature API, fenofibrate, with HPMC-AS as a polymeric carrier by adjusting the drug loading and the processing temperature of the HME process (38). Fenofibrate seems to have a plasticizing effect on HPMC-AS; for example, only when the drug loading is high (30%) can HME be carried out at a lower temperature of 90°C. However, to achieve better ASD stability and improved drug release, the optimum drug loading is lower (15%). At low drug loading, the minimum required extrusion temperature remained at 130°C. Fan et al. reported an optimized HME process for the preparation of curcumin sustained-release ASD (58). The study showed that curcumin existed in an amorphous state in the ASD and had molecular interactions with the polymeric carrier. The curcumin was released *via* a combination of diffusion and dissolution from the polymeric matrix.

As HME has been widely studied for the preparation of ASDs, it was reported that high melting points API (*e.g.*, those drugs having a melting point > 200°C) generally can be more difficult to process *via* HME, as the high processing temperatures can present challenges of chemical instability to the API mixture and can cause thermal degradation to the API or polymer. There are several methods suggested to overcome this issue of the high processing temperature. One of the methods is to suppress the melting point of the API by mixing it with a favorable polymer, in which the API can dissolve in the polymer when it melts. The polymer must become molten at a lower processing temperature and dissolve the API. Guo et al. demonstrated the use of the API-polymer interaction technique to prepare a chemically stable ASD *via* HME (59). In this study, diflunisal (DIF) was used as the model API and the interaction of DIF with four different polymers (PVP VA64, soluplus, hydroxypropyl methylcellulose (HPMC), and PVP K30) were investigated. The study showed that the API had good miscibility with the polymers and that when the polymer was present, the ASD processing temperature was much lower than the melting point of DIF in its crystalline state. In the stability study, all API-polymer combinations apart from DIF-HPMC were stable and maintained its amorphicity up to 3 months in an environment of 40°C and 75% RH. The physicochemical stability of the ASDs was attributed to the hydrogen bonding formed between DIF and the polymers.

Another common method for lowering the processing temperature is by adding a compatible plasticizer or surfactant to solubilize the API and the polymer at lower temperatures (60). The study by Zhang et al. successfully lowered the HME processing temperature to produce an ASD of baicalein (BAC), with the use of a plasticizer, Cremophor RH, to improve the bioavailability of the API (61). The plasticizer was able to soften the polymeric carrier and decrease the glass transition temperature as well as the melt viscosity of the polymeric carrier, allowing BAC to solubilize at a lower temperature. In addition to improving the API’s dissolution and bioavailability, the study showed that careful selection of a compatible plasticizer improves the processability of a high melting point API *via* HME. Zhao et al. also studied the effect of plasticizers on the ASD of ritonavir as API and copovidone as a polymeric carrier produced *via* HME (62). The study investigated the impact of two different plasticizers (Span 20, hydrogenated soybean phosphatidylcholine (HSPC)). The results showed that the plasticizers are effective in not just in lowering the extrusion temperature, but also in improving the dissolution profile and bioavailability of the ritonavir ASD.

### Pharmaceutical Co-crystals

Due to the challenges and stability challenges of ASDs, researchers have discovered other systems that similarly enhance the solubility and bioavailability of poorly soluble APIs. As APIs in crystalline state are generally more stable than in an amorphous state, the development of pharmaceutical co-crystals has emerged as an attractive alternative for solubility enhancement. Co-crystals are solid, neutral, crystalline materials containing two or more different molecular/ionic compounds in a stoichiometric ratio, where the compounds are held together *via* non-covalent forces such as ionic interactions, hydrogen bonds and Van der Waals forces (63,64). An active pharmaceutical ingredient (API) and a co-former can make up a pharmaceutical co-crystal. The API and co-former typically interact *via* non-covalent bonding, and notably, there is no proton transfer between the API and co-former.

For this reason, co-crystals have the potential to be applied to all APIs, including acidic, basic, and non-ionizable molecules (65). The purpose of co-crystals is to improve the solubility and bioavailability of the API without affecting the physiological action and compromising the structural integrity or thermal stability of the crystalline API (66). Apart from that, it is reported that the mechanical properties of the API can be improved in co-crystals, which is highly beneficial for the preparation of dosage forms. The new product formed between an API and co-former most certainly has a different melting point; that could be lower, higher, or any point in between the melting points of the original compounds. Co-crystals can form a solid dispersion during the preparation process, but not all co-crystals are solid dispersions. Some of the pharmaceutical co-crystals currently available in the market include ipragliflozin-proline (Suglat, Astellas Pharma, and Kotobuki Pharmaceutical) and valsartan-sacubitril (Entresto, Novartis) (45,67).

To date, co-crystals have been prepared using solid-state techniques such as grinding (68) and ultrasound sonication (69–71), or *via* solvent evaporation methods (72). However, the main challenge in these techniques is the difficulty in scaling up for mass production. When using the solvent-based techniques, the components used must have similar solubility to prevent precipitation and dissociation. Co-crystals formed from compounds with different solubilities can cause co-crystal dissociation, recrystallization of API, and partial dissolution of co-formers (73,74). Such formulations will potentially reduce the solubility and bioavailability of the API (75). Therefore, extensive knowledge of the ternary phase, between the materials, for the co-crystals formation and the solvent is required. To obtain such information for the creation of phase solubility diagrams, a series of experiments are necessary, which requires much effort and can be very time consuming (76,77). Even though there have been efforts in simplifying experimental procedures for the scaling up of co-crystals production *via* solution crystallization, it is still quite a complex process (78,79). When solvents are used for crystallization, the solvents present in the co-crystals need to be dried to an acceptable level as excessive solvents and impurities can be harmful and cause toxicity (80,81). Due to the complexity of solvent-based techniques, solid-state synthesis of pharmaceutical co-crystals is often preferred as it is more efficient to modify the physicochemical properties (such as melting point, solubility, compressibility, stability) of the API (82–85). Solid-state techniques include grinding methods such as neat grinding in mills, liquid assisted, or polymer assisted grinding (86–88). The grinding methods eliminate the need for the knowledge of the solubility of the co-crystal materials. They can be considered a green method as it eliminates the use of solvents (89). However, scaling up of the grinding methods for large-scale production of co-crystals is also a limitation.

HME was then employed for co-crystals production to overcome the limitations of the traditional co-crystals production methods. In addition, HME, in particular twin-screw extrusion, can provide homogenous and efficient mixing as the materials are closely packed together in the barrel. At the same time, the rotating screws improved the surface contact between the individual components through shear mixing, facilitating the formation of co-crystals without the presence of solvents (90). Medina et al. are the first to report of co-crystallization production using twin-screw extrusion (91). The efficient and homogenous mixing of twin-screw extrusion can effectively facilitate the formation of co-crystals. Dhumal et al. demonstrated how the solvent-free process of HME is scalable for the production of Ibuprofen-Nicotinamide co-crystals in the agglomerated form (92). The study also investigated the effect of processing parameters of HME, such as the temperature, screw configuration, and residence time. It was reported that processing above the eutectic point is required for the formation of co-crystals (92). Other reports showed that the screw design, temperature, feed rate, and screw speeds are the primary factors affecting the conversion of co-crystals (85,93,94). However, the barrel temperature in HME is still the most influential factor for co-crystal formation in the process (85,95,96). Daurio et al. managed to produce a wide variety of co-crystals, including caffeine-oxalic acid, AMG 517-sorbic acid, theophylline-citric acid, carbamazepine-saccharin, *etc.*, using the twin-screw extrusion technique (80,90). One of the studies compared the co-crystals of AMG 517-sorbic acid produced using twin-screw extrusion and standard solution crystallization, showing the extruded co-crystals have superior mechanical properties than solution grown co-crystals (80). The feasibility of the liquid-assisted HME process using benign solvents (such as water or ethanol) as a catalyst for the formation of co-crystals was also investigated (90). The researchers demonstrated that the presence of benign solvents promotes co-crystal formation at lower temperatures.

Although APIs are generally more stable in their crystallized form, pharmaceutical co-crystals have physical stability issues that are associated with the APIs tendencies to form hydrates in the presence of water molecules. Hydrate formation is associated with the small particle size and the multidirectional hydrogen bonding abilities of the co-crystals, particularly at higher temperatures and humidity when the co-crystals absorb moisture from the atmosphere. API in its hydrated or anhydrate form is generally unstable (65). An innovative approach, known as the matrix-assisted-co-crystallization (MAC), was developed to improve the chemical and physical stability of co-crystals (97). Apart from that, MAC minimizes the stress exerted by the rotating screws onto the solid crystalline compounds during the extrusion process, which could also prevent excessive wear and increased energy consumption by the extruder (98).

Matrix-assisted co-crystals (MAC) is a novel co-crystal synthesis method, where equimolar quantities of the API and co-former materials are embedded into a polymeric matrix in the solid-state before the hot-melt extrusion process. During the extrusion process, only the matrix material needs to be melted or softened. Similar to a conventional HME process, the mixture will solidify upon exiting the extruder, and the extrudates or products are hereafter known as matrix-embedded co-crystals. Co-crystallization of the API and co-former occurs in the melted/softened polymer matrix during the extrusion process, allowing the intimate mixing of the individual components. The polymer matrix serves two roles in the MAC. During the HME process, the matrix acts as a catalytic solvent as the molten matrix promotes for the formation of co-crystals by creating intimate mixing while preventing excessive shear stress exerted onto the solid materials by the rotating screws. Excessive shear stress may cause damage to the solid crystals, which may cause chemical and physical stability issues. In the product, the matrix acts as a functional component of the formulation, which can affect the physicochemical properties of the final formulation, such as the flowability, compaction, and API release kinetics. Boksa et al. reported that the MAC synthesis method is useful in producing high-quality co-crystals (99). In this study, carbamazepine, nicotinamide, and soluplus were used as a model drug, co-former, and matrix, respectively.

Karimi-Jafari et al. have also verified the advantageous effect of the presence of excipients in the co-crystal sample (94). Co-crystals of ibuprofen-nicotinamide were prepared using HME, and soluplus was added as the polymeric excipient. It was shown that the presence of soluplus decreased the co-crystallization temperature as its presence enhances the interaction between the API and co-former. The study also proved that the mechanical and tabletting properties (tabletability, compactibility, and compressibility) were significantly improved with the presence of soluplus when being compared to purely ibuprofen-nicotinamide co-crystals only (94).

Ross et al. also reported a similar method of preparing MAC, in which the API and co-former (indomethacin-saccharin) blend were co-processed with an inert and non-miscible excipient (100). In this study, three different excipients (a crystalline hydrophilic polymer PEG 6000, an amorphous hydrophilic polymer hydroxypropylmethylcellulose, HPMC, and an aluminometasilicate inorganic excipient Neusilin) were used, and the effects of these polymeric excipients on the co-crystals were compared. The excipients were added into the extruder conveying zones after co-crystallization of the API and co-former occurred, causing the co-crystal particles to be embedded into the molten excipient. The results showed that the HPMC matrix was able to slow the dissolution rates, but Neusilin and PEG 6000 did not affect the dissolution rates of the co-crystals. In general, the co-crystals showed enhanced physicochemical stability when embedded into a polymeric matrix.

The work reported by Shaikh et al. showed the successful production of theophylline-4-aminobenzoic acid (4ABA) co-crystals with PEG, as a hydrophilic excipient using HME (95). The extrudates showed superior tabletting performance when compared to pure theophylline-4ABA co-crystals; additionally, the co-crystals were stable up to 14 days even in high temperature and humidity (50°C and 75% RH). The study also investigated the effect of PEG grades on the quality of the co-crystals formed, showing that there was no pure co-crystal formation with PEG 1500. On the other hand, higher M.W. PEG 8000 showed the formation of pure co-crystals. It seems that the components were solubilized into the lower M.W. PEG, whereas higher M.W. PEG is less miscible due to the reduced Gibbs free energy of mixing. The concentration of PEG in the co-crystals formulation can affect the quality of co-crystals as well. It is reported that the concentration of PEG should not be more than 5% as higher concentration leads to less pure co-crystals production, in which the higher concentration of PEG may restrict the molecular collision between the API and co-former (95).

The idea of using polymeric excipients that are inert, non-miscible with the compounds forming the co-crystals to obtain high-quality MAC, was introduced by Li et al. (101). The inert excipient prevents any strong interaction between the polymer matrix and the co-crystals that could potentially affect the yield of the co-crystals. Therefore, the careful selection of the polymeric matrix is essential. Gajda et al. then investigated the effect of different types of polymeric matrixes, analyzing a wide range of polymers that exhibited various structural features and physicochemical properties, to better understand their role in MAC (102). The model co-crystals formulation flufenamic acid-nicotinamide (FFA-NA; 1:1) was mixed with five different polymers (Poloxamer P407, PEG-PVA copolymer, soluplus, PVPVA64, and HPMC-AS) to form MAC. The study showed that the dissolution of FFA was improved when embedded in semicrystalline polymers, poloxamer and PEG-PVA, when compared to pure FFA-NA co-crystals. The research suggests that semicrystalline polymer, regardless of its melting temperature, may promote co-crystallization. The amorphous polymer (soluplus, PVPVA64, HPMCAS) resulted in the formation of FFA-NA co-crystal embedded in an amorphous matrix, essentially an amorphous solid dispersion, when the polymer concentration was up to 20% for HPMCAS and up to 30% for soluplus and PVPVA64. Ultimately, the presence of polymer matrix decreased the torque value required for the processing which the throughput of the process may be increased (102).

Butreddy et al. produced co-crystals of aripiprazole (ARP) and adipic acid (ADP) *via* a solvent-free HME process. This study investigated the effect of Soluplus, the processing temperature, and the screw speed on the ARP-ADP co-crystal (103). Interestingly, by incorporating 5% Soluplus within the process, the extrusion process no longer exceeds the maximum torque limit and can now successfully process the co-crystals. The presence of Soluplus improved processability and facilitated the interaction between ARP and ADP to form co-crystals; as mention previously, Soluplus has successfully been used as a polymeric excipient for other co-crystals formation (82,94). The study again proves that extrusion temperature is critical for the successful formation of co-crystals. The produced ARP-ADP co-crystals show an 8-fold improvement in solubility and a 7-fold improvement in dissolution rate compared to the pure ARP.

### Other Recent Innovation Formulations *via* Hot-Melt Extrusion

The versatility of the HME technology has allowed researchers to explore its capability in producing other formulations for solubility and stability enhancement. Some of the recent innovations for HME prepared formulations include co-amorphous system, co-extrusion systems, and semi-solid formulations. A co-amorphous system has shown some promising results for solubility enhancement in the delivery of poorly water-soluble drugs. A co-amorphous system can be defined as a single phase system made up of a crystalline API and a co-former of low molecular weight molecules which could be either relevant APIs or excipients (104). Some examples of the co-former include urea, sugar, amino acids and carboxylic acids, which are known to be able to enhance the stability and solubility in a co-amorphous system (105). The co-amorphous system has gained much attention as studies have shown that it is capable of overcoming the issues often seen in ASDs such as poor stability, poor drug solubility with polymers, phase separation and recrystallization due to the hygroscopic nature of polymers. In a co-amorphous system, the drug is incorporated with small molecules instead of polymers. Hence, co-amorphous systems can provide dissolution enhancement and stability advantages. The commonly used techniques for the preparation of co-amorphous systems include ball milling (106), cryo-milling (107), solvent evaporation (108), and spray drying (109). Due to the limitations of scaling up for these processes, the feasibility of producing co-amorphous systems *via* HME has been explored. Similar to the preparation of other drug delivery systems, HME for co-amorphous systems offer the advantages of easy scale-up and in-line monitoring of the materials to ensure quality and prevent degradation. The continuous manufacturing of co-amorphous system using HME was first introduced by Lenz et al. for the development of indomethacin-arginine (IND-ARG) co-amorphous system *via* a twin-screw extruder. The study showed that IND-ARG system had an improvement in the dissolution with an additional polymer (copovidone) and the stability was comparable to the one produced *via* spray drying. However, reports on the HME prepared co-amorphous systems are still very limited in the literature as phase separation can occur in such systems and the polymers suitable for use in the HME manufacturing of co-amorphous systems are limited due to rheological issues (105,110).

Hot-melt co-extrusion is a relatively new technique applied in the pharmaceutical industry. This technique can create a multilayer extrudate consisting of two or more materials simultaneously in a single extrusion process. The advantage of this technique is that it could combine APIs with different release pattern in a single dosage form, allowing the incorporation of different drugs in a single dosage form for patients (111). The concurrent extrusion to obtain a multi-layered extrudate can be achieved by using two extruders with a co-extrusion die attached. Dierickx et al. have successfully developed a multi-layered dosage form by using one drug, diclofenac sodium, to achieve two different release profiles *via* the co-extrusion technique (112). This was achieved by incorporating the drug with polymeric excipients with different properties, one lipophilic and the other is hydrophilic. During the co-extrusion of a multilayer extrudate, it is also important to ensure the adhesion between each layer of the extrudate. Therefore, the selection of suitable polymers is important. Vynckier et al. reported the production of a sustained release dosage form containing two antidiabetic drugs with different solubility—metformin and gliclazide, *via* the co-extrusion technique using an HME (113). The two drugs have been extruded to form a single dosage form incorporated with polymeric excipients that could sustain the release of both drugs. The highly water-soluble metformin has been incorporated as the core and the poorly water-soluble gliclazide was incorporated as the coat for the monolithic dosage form. The results from the dissolution study showed that the bilayer fixed-dose combination product exhibit a sustained release profile up to 24 h for both drugs. The use of combined therapy for diabetes has been reported to have better control of the glucose level as compared to just a single drug therapy (114). The study showed that the co-extrusion technique is perfectly suitable to combine different APIs with different physicochemical properties into a multilayer single dosage form. The biggest challenge of co-extrusion is the selection of suitable polymers. With the selection of suitable polymeric matrix, the co-extruded single dosage form can achieve a desired release profile even though the solubility of the drugs are incompatible to each other.

HME has also been successfully used to develop semi-solids such as ointments, gels, nanostructured lipid carried gels, and creams (115,116). HME is often more effective compared to tradition semi-solid preparation methods that are more time consuming as HME carries out melting and mixing of the materials in a single automated process (117). However, there is still very limited studies being carried out on using HME for the preparation of topical semi-solid formulations. Bhagurkar et al. have been one of the firsts to report the use of HME for the preparation of semi-solids formulation (117). The study successfully prepared a polyethylene glycol-based ointment, using Lidocaine as an API *via* HME with a modified screw design. The screw contains three mixing zones for better mixing and uniformity in the extruded ointment. The first mixing zone is to ensure the proper mixing of Lidocaine and PEG. The second mixing zone is to provide more intense mixing of the different components to ensure homogenous mixing. The third mixing zone is to prevent the agglomeration of the mixed components before extrusion. In this study, optimization on the processing parameters such as feeding rate, barrel temperature, and screw speed was required to obtain a uniform product. The ointment produced *via* HME were smooth and contained uniform drug content. The quality and properties of the HME produced ointment were comparable to the one produced *via* conventional techniques. However, HME offers the advantage of having minimal processing steps and better efficiency. Thakkar et al. has successfully shown the possibility of using HME to produce topical semi-solid formulations using two different APIs—triamcinolone acetonide (TAA) and lidocaine hydrochloride (LDH) (118). The study has shown that LDH improves the stability of TAA which could improve the shelf life of the dosage form. Such a formulation with two different APIs could provide an additive effect in the treatment of aphthous ulcers and pruritus (119).

Due to the cost-effectiveness and the ability of the HME technology to scale up for the industrial application, HME has a great potential to be used for the production of many other drug delivery systems. Sarabu et al. has published an update on the contribution of HME to some of the most recent and novel drug delivery systems, including those mentioned above (120).

## RECENT INNOVATIONS IN HOT-MELT EXTRUSION TO PREVENT THERMAL AND CHEMICAL DEGRADATION

High melting point APIs whose stability is primarily compromised by thermal degradation (i.e., thermally labile) compared to degradation driven by excipient incompatibilities (i.e., chemical instability) during the HME process experience the most significant benefit from decreasing the processing temperature. Contrarily, for molecules that experience chemical instability (*i.e.*, amide or ester hydrolysis) during HME processing, reducing the processing temperature is not always sufficient to achieve a viable ASD (121,122). For molecules experiencing chemical instability during thermal processing, a fundamental understanding of the degradation pathway is essential in creating an ASD without trace crystallinity and acceptable degradation products. Hydrolysis is the most common chemical degradation pathway; two of the most susceptible groups are esters and amides (123). Ester hydrolysis occurs at a faster rate than amide hydrolysis, which is attributed to the differences in electronegativity between the functional groups (124).

Moreover, amorphous state degradation kinetics significantly increases compared to that of the solid-state, where the amorphous state experiences degradation kinetics similar to an API in solution (122). Additionally, acidic or basic environments further promote degradation *via* acid or base-catalyzed hydrolysis of the ester or amide present. All of the pathways mentioned above are temperature dependent; therefore, prolonged times at elevated temperatures will increase degradation when an API is in combination with excipients that promote degradation (121,125). Though decreasing the temperature in these circumstances will reduce degradation (121,122,125), it is not the underlying cause.

### Specific Energy Impacts Crystallinity and Degradation

The total energy input into a system is dependent on both the specific mechanical energy (SME) and thermal energy input from the extruder. Modifying variables that influence these energy inputs (*i.e.*, barrel temperature, screw RPM, feed rate) enables the creation of design space to form extrudable regions where degradation is eliminated, and crystallinity is absent (122,126). Though optimizing the energy input and screw design significantly improves degradation profiles, it is not always effective in overcoming degradation, therefore, requiring other innovative HME techniques to overcome degradation.

### *In Situ* Salt Formation Prevents API Degradation

**Fig. 6:**
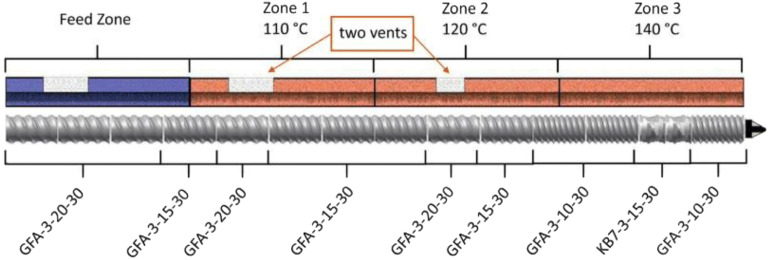
Optimized barrel and screw designs to prevent degradation during the extrusion process of converting meloxicam to its amorphous form (adapted with permission from ref. (121))

When compositional components promote degradation, process optimization can only take the formulation so far; for example, Haser et al. optimized process parameters to increase meloxicam (MLX) purity from 79 to 98%. In this study, they determined residence time and temperature significantly impacted MLX degradation. Despite this knowledge, process optimization was unable to eliminate the 2% degradation present (Fig. 6). It was not until a forced degradation study revealed that MLX was stable in basic conditions and degraded in acidic conditions, that the researchers were able to eliminate the degradation. Copovidone, the polymer used in the meloxicam study, contains acetyl groups, decreases the microenvironment pH of the composition, promoting MLX degradation *via* hydrolysis (121). Understanding the polymer effects on the microenvironment pH and its impact on MLX degradation, the pH modifier meglumine, formed a salt between its secondary amine and the anionic carbonyl present on MLX, stabilizing the composition and eliminating degradation (121,127).

### Venting and Solvent Assist to Prevent Hydrolysis

Prior to incorporating meglumine, in an attempt to eliminate degradation by hydrolysis, Haser et al. sought to reduce moisture during the extrusion process by venting the extruder, resulting in no change to the degradation profile (Fig. 6). Notably, the authors suggest that the closed-vent condition had an inflated purity value due to the retained water plasticizing the composition. This decreased the melt viscosity to an extent where more aggressive mixing conditions were needed for amorphous conversion (121). On the other hand, Evans et al. used vented HME to develop soluplus:torasemide compositions as an attempt to minimize API hydrolysis as torasemide is sensitive to both thermal and hydrolytic degradation (128). Surprisingly, upon venting, the degradation was maximized. Counterintuitively, the moisture was acting as a plasticizer; when removed, the viscosity increased, increasing the viscous dissipation, which ultimately increased degradation products from the increased melt temperature (128). If venting is adapted in a process, further process optimization is required to account for the change in melt viscosity with water loss. For example, Hengsawas et al. explored the effect of changing process parameters on albendazole utilizing a venting site through the entirety of the study. In this study, venting was not sufficient to prevent the base-catalyzed hydrolysis; therefore, the authors incorporated a solvent assist technique to decrease the operating temperature, increasing purity 11-fold (15). However, albendazole degradation was still greater than 70%; therefore, the authors deemed HME not a suitable process for albendazole; future attempts may require stabilization *via* a ternary component to prevent rapid base-catalyzed hydrolysis of the amorphous state.

### API-Polymer Incompatibilities Promote Degradation

Polymer selection should not only be dictated by its miscibility with an API; equally important is the polymer’s ability to prevent degradation. Further emphasizing the importance of understanding an API’s degradation pathway, Wagner et al. utilized polyvinyl acetate phthalate (PVAP) with dipyridamole (DPD) to unsuccessfully produce HME ASDs. Upon process optimization, the purity profile was improved from 0.8 to 7.5%; the study concluded PVAP was not a suitable carrier for DPD (129). In the extrusion process, phthalate acid, an acidic moiety, is generated. From the molecular structure, DPD contains four primary hydroxyl groups that are highly susceptible to acidic environments, knowledge of this would predict incompatibility with a polymer containing acidic groups. For example, DPD has been successfully melt-quenched and extruded using both HPMC E50 and copovidone, respectively (130,131); therefore, overcoming DPD degradation required a fundamental understanding of API-polymer compatibility to select an appropriate polymer that would not promote degradation. In a study utilizing spray drying, DPD with Eudragit S100 produced an ASD with no degradation, the addition of 15% tartaric acid resulted in a 20% decrease in purity (132,133).

#### Hydrate Formation Decreases Processing Temperatures

Creative strategies have emerged, showing promise as innovative formulation approaches to eliminate degradation products when formulating ASDs. Ma et al. intentionally converted anhydrous carbamazepine to the dihydrate form by mixing anhydrous carbamazepine in distilled water for 48 h. The solids from the suspension were obtained through filtration and were dried by lyophilization, forming carbamazepine dihydrate. By utilizing the desolvation energy of the hydrate, an amorphous solid dispersion can be formed at decreased processing temperatures. Differential scanning calorimetry determined desolvation occurred from 40°C to 80°C and recrystallization occurred at 84°C (134). During the dehydration process of carbamazepine dihydrate, the carbamazepine molecules can only stay disordered at low temperatures, which would subsequently recrystallize to its anhydrous form at higher temperatures. Therefore, to utilize the desolvation energy and prevent recrystallization, soluplus:vitamin E succinate was melted at 140°C in the first two zones, and then carbamazepine dihydrate was fed in at the third zone and processed at 60°C. Additional energy input provided by a more aggressive mixing using a 90-degree mixing element, compared to a 60-degree mixing element, was able to completely convert carbamazepine into an amorphous solid dispersion using HME at 60°C. The carbamazepine dihydrate also exhibits better miscibility with the polymer matrices, allowing the formation of carbamazepine ASDs at lower temperatures (134). More than one-third of APIs have the capability to form hydrates (135), so applying this approach to APIs that have an increased propensity to degrade at elevated temperatures is potentially a means to circumvent degradation by processing at decreased temperatures.

#### In-line Process Monitoring Tools to Improve Processing Parameters

The pharmaceutical industry has been moving towards continuous manufacturing not just to maximize the manufacturing efficiency and economic benefits, but also to fulfill the requirements of regulatory bodies. Therefore, the shift to continuous manufacturing in the pharmaceutical industry is initiated by the introduction of QbD by the FDA (136). QbD is a systematic approach for pharmaceutical development and manufacturing by emphasizing the product and process understanding and process control based on sound science and quality risk management (137). In general, QbD implementation aims to ensure product quality and production efficiency during the pharmaceutical product development process (136). HME is a versatile and robust continuous manufacturing method that has become an essential pharmaceutical manufacturing technology. In order to further optimize the maximum efficiency of the HME process for pharmaceutical product development and manufacturing, a thorough understanding of the effect processing parameters on the product is required. Hence, there has been much effort in introducing PAT tools to be incorporated within the HME process in order to achieve in-line process monitoring. Traditional off-line analytical techniques, which are mainly carried out on intermediate and end products, are time-consuming and do not give an accurate representation of the quality of the product produced through continuous manufacturing. Real-time monitoring can allow the optimization of the process parameters to achieve the desired output during the process. During the HME process of pharmaceutical products, information such as the API solubility, crystallinity, stability, and content uniformity in the mixture need to be closely monitored to ensure the product quality and how different processing parameters may influence the process (138). Therefore, in-line PAT is also beneficial for new product design and development as it allows fast product screening to design a stable system that has maximum therapeutic efficiency.

Some of the most commonly used PAT tools include UV-Vis (139,140), Fourier transform near-infrared (FT-NIR) (104,119), and Raman (141,142) spectroscopy techniques. These techniques are simple, fast, and non-destructive to the samples during the HME process. FT-NIR can provide information on the molecular interaction of the materials, such as the API and its polymeric excipients. A technical note by Vo et al. showed the feasibility of incorporating FT-NIR as an in-line monitoring tool to monitor the API concentration during the HME process (143). The study showed the in-line NIR was able to provide chemical information such as the blending ratio and interactions between the API and polymer, as well as physical properties of the sample such as color, temperature, and density. As NIR is a non-destructive and fast monitoring tool, it can also be used for both product development and manufacturing (143).

Islam et al. used in-line NIR to optimize the development of paracetamol sustained-release formulations during the HME process (144). The in-line NIR was used as a PAT to investigate the effect of screw speed, feed rate, and drug loading on the dissolution rate and particle size distribution of the mixture during the HME process. Principal component analysis (PCA) of the NIR spectra collected was used to obtain information on the optimum processing parameters. The study showed that drug loading has a significant influence on the dissolution rate. Moradiya et al. demonstrated the coupling of NIR monitoring with HME as an in-line PAT to monitor the formation of carbamazepine-trans-cinnamic acid co-crystals during the HME process (85). Fiber optic NIR probes were placed across the three different zones along the extruder barrel, which showed the gradual development of co-crystals through the formation of hydrogen bonding. The study also compared the quality of co-crystals formed from single-screw extrusion and twin-screw extrusion, showing the twin-screw extruded showed faster dissolution rates, which could be the increased contribution of dispersive and distributive mixing in the twin-screw extrusion process.

Apart from NIR, Raman spectroscopy is also a very effective PAT to provide valuable information regarding the quality of the product during the HME process. Saerens et al. carried out a study to evaluate the suitability of Raman spectroscopy to be used as an in-line monitoring PAT during a pharmaceutical HME process (141). The Raman probe was placed in the die to measure the different concentrations of metoprolol tartrate (MPT) in Eudragit® RL PO. The prediction from the Raman was obtained from using the Partial Least Squares (PSL) model *via* the regression of MPT concentrations *versus* the Raman spectra. The results showed that the correlation between the predicted and real MPT concentration of the samples is acceptable. The feasibility of using Raman spectroscopy for solid-state characterization on the extrudates was evaluated. Raman spectra of two different concentrations of the API-polymer mixture showed different peaks, which suggested a difference in amorphicity and interactions between the mixtures. The observations from Raman were further confirmed with DSC analysis. Hence, the study has proven that Raman spectroscopy can be used as an in-line PAT to effectively determine API concentration and polymer-API solid-state characterization during the HME process (141).

Bordos et al. proposed a novel approach of using an in-line terahertz (THz)-Raman Spectroscopy as a PAT to determine the saturation point of an API in a polymeric matrix during the HME process (145). The study carried out here uses paracetamol as a model API dispersed into two polymer systems, HPMC and copovidone. The results obtained from the THz- Raman showed the transition of API from the crystalline state to the amorphous state in the polymeric matrix. The results were used to construct the solubility phase diagram of the system to show the solubilization capacity of the polymeric systems. The study discovered that the ASD of paracetamol has maximum stability when its concentration is 20 wt% in HPMC and 40 wt% in copovidone. The study showed that this novel spectroscopy combination can be used to provide real-time API-polymer phase equilibria and can be used to predict the stability of ASDs during the HME process (145).

The work carried out by Tahir et al. showed an innovative approach to implementing process monitoring and fault detection onto a pharmaceutical HME process (146). The HME process monitoring was achieved by an in-line Raman spectroscopy; whereas, the fault detection scheme can be realized by the incorporation of a hybrid soft sensor (147). The system was designed to monitor the drug concentration during the extrusion process of the paracetamol-affinisol extrudate, as well as identifying operational faults during the HME process, accomplished by using the two sensors independently. The drug concentration in the extrudates was predicted using the partial least squares (PLS) model developed by regressing the Raman spectra. The API concentration prediction data were then fed into the PCA and statistical process control (SPC) monitors to detect some of the HME operational faults such as zone heater failures, feeder failure, and Raman probe faults (146).

**Fig. 7:**
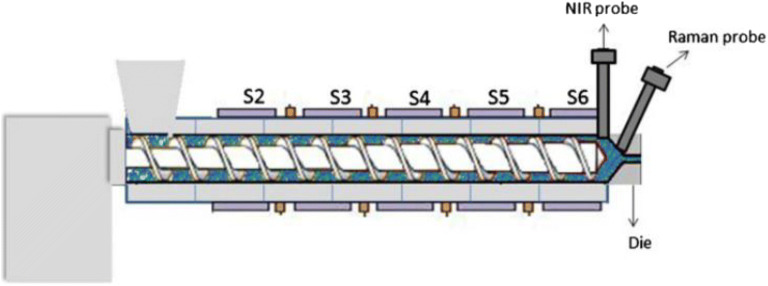
Schematic diagram of an HME incorporated with in-line NIR and Raman as PAT (adapted with permission from reference (148))

A study carried out by Almeida et al. implemented two spectroscopic techniques for the in-line processing monitoring of ethylene vinyl acetate formulations prepared using HME. The extruder was coupled with both in-line Raman and NIR spectroscopy to investigate the effect of barrel temperature and screw speed on the materials at a molecular level during the extrusion process (Fig. 7) (148). The information provided by the in-line PAT tools includes the solid-state and the molecular interactions of the drug with the polymeric matrix as a function of the process paraments. Such information allows the effective up-scaling of the HME process from a lab-scale extruder as it enables a better understanding of the molecular interactions in the materials during the extrusion process. The information also allows the user to adjust the temperature and the screw speed accordingly to achieve the desired output. These studies have shown the versatility of the HME process in which the technology can be integrated with other monitoring tools to maximize production efficiency and achieve the best formulation outcome.

In addition, an in-line UV-Vis spectroscopy has been developed by Schlindwein et al. to be used as a monitoring tool for experimental designs of ASD preparation *via* HME, which is particularly useful for early phase product development (140). The in-line UV-Vis can be used to assess the solubility of an API in the polymer. The use of in-line UV-Vis during the extrusion process allows a better understanding of the effect of processing parameters such as the die temperature, screw speed, feed rate, and API concentration on the mixture. A further study on the use of UV-Vis for measuring API concentration in the polymer and API content uniformity was carried out. Analytical models based on the UV-Vis spectra obtained during the HME process were developed as a quantitative method to predict the concentration and content uniformity of piroxicam in Kollidon® VA 64 (139). The study by Haser et al. showed the possibility of in-line monitoring of the solubilization of meloxicam using a UV probe positioned at the die (149). UV-Vis can only provide limited chemical information, such as molecular interactions, when compared to NIR and Raman. Nevertheless, UV-Vis is quick and non-invasive for reliable early product development. This process is also non-invasive and does not interrupt the continuous process.

#### KinetiSol® Dispersing

As mentioned previously, the well-known limitation of thermal processing in pharmaceutical applications, in particular the HME process, is being able to process at elevated temperatures for prolonged periods, which can significantly compromise the integrity of thermolabile APIs and excipients. Typically, high melting point APIs (> 200°C) require high processing temperatures (121). On the other hand, most polymeric carriers have lower melting points and glass transition temperatures. Therefore, high-temperature processing can cause thermal degradation and has ultimately limited the choices of APIs and polymeric excipients suitable for HME (150). Although the introduction of plasticizers into the composition can lower the processing temperature of high melting point APIs during the preparation of ASDs *via* HME, the stability of ASDs can be compromised. Therefore, an innovative technology, KinetiSol® Dispersing, has emerged as a promising technique for the production of ASD without the need for plasticizers.

**Fig. 8:**
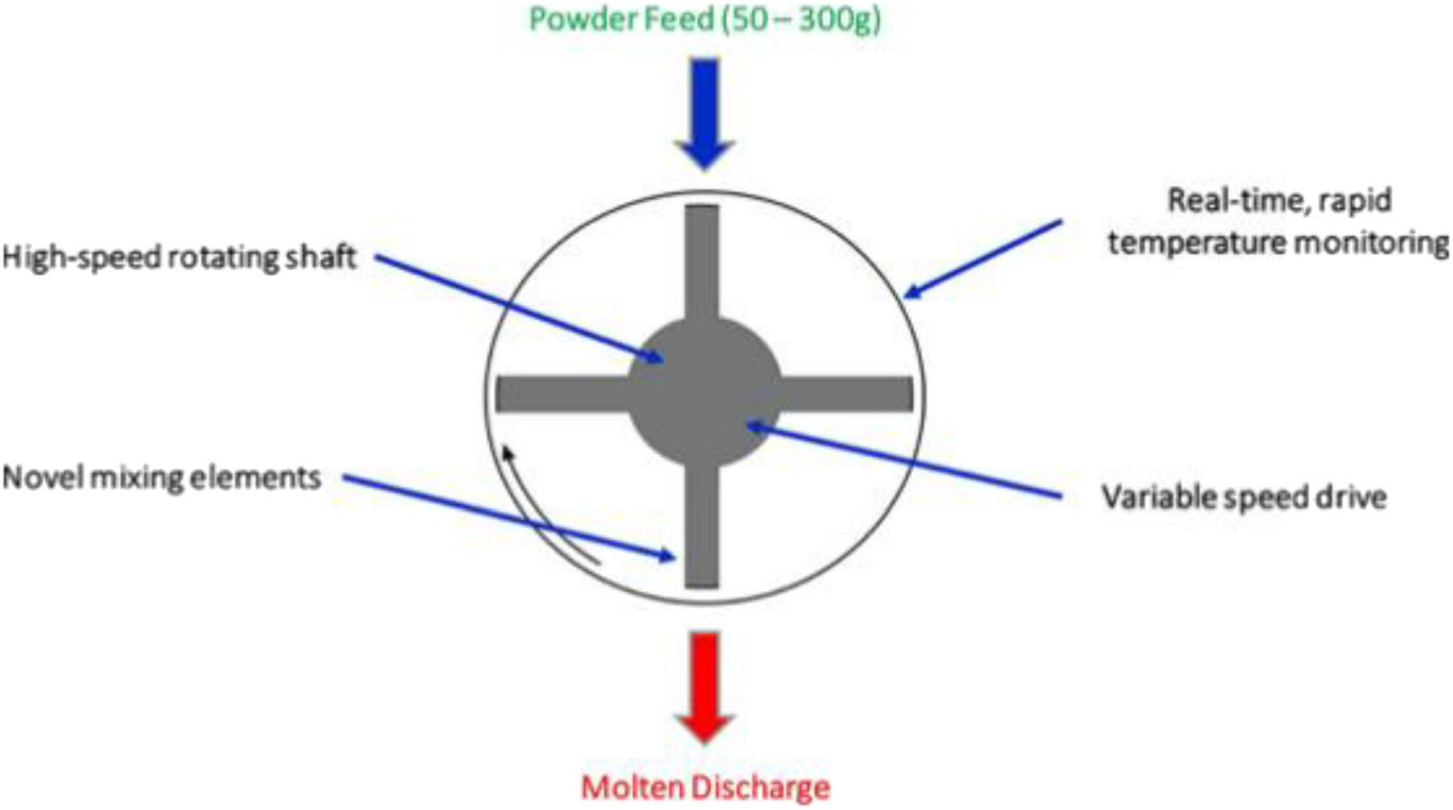
Simplified Schematic diagram for a KinetiSol Compounder (reproduced with permission from (8); adapted)

Specifically, KinetiSol® dispersing (KSD) technology is one of the newer thermal processing techniques that was invented to manufacture ASDs *via* the high-energy fusion-based method (18,151). Although the pharmaceutical applications of this technology are relatively new, compared to HME, it can overcome many of the limitations for the preparation of ASDs. Adapted to fit the demands of pharmaceutical applications, KSD technology is similar to the thermokinetic mixers used in the plastics and recycling industry. The KSD equipment has a chamber with a horizontal rotating shaft at the center instead of a set of rotating screws. A series of mixing blades are attached to the rotating shaft, which rotates at very high speeds (> 1000 rpm) (Fig. 8 (18)).

**Fig. 9:**
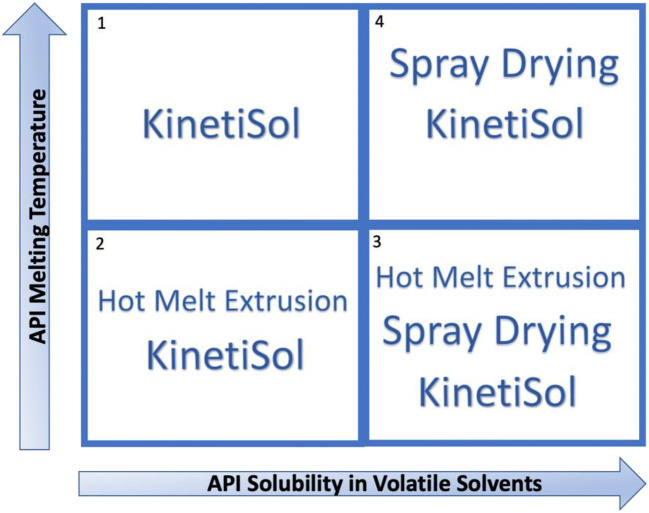
Amorphous solid dispersion applicability matrix in response to API properties (adapted with permission from (8))

The blade’s rapid speed forces the composition in the chamber to rub against one another and impact with both the walls of the chamber and blades, creating intense frictional forces. As a result, a rapid increase in the compositional processing temperature occurs. Unlike HME, there is no heating element to provide external heating to the chamber. The heating is solely induced by the mechanical shear and frictional forces created during the process. Processing times are brief, typically only less than 20s, with the material usually exposed at elevated temperatures for less than 5 s before the compositions are ejected and quenched (22). In addition, the temperature in the chamber while processing is typically well below the melting point or the degradation temperature of the compounds (152). The short processing times, low processing temperatures, and high mixing intensity offer many advantages, particularly for pharmaceutical applications. The technology is capable of processing highly viscous, non-thermoplastic materials as there are no torque limitations, hence allowing a more extensive choice of polymers to be used. As such, the use of plasticizers can be eliminated (153). KSD is also capable of processing highly thermally sensitive APIs or high melting point APIs into an ASD using quite short processing times. KSD applications have been primarily limited to the production of ASDs, as the technology was designed to overcome limitations of HME. To date, expanding KSD applications for the production of co-crystals, co-amorphous, semi-solids, and co-extruded multilayer systems have not been reported in the literature and warrants further investigation. Figure 9 is a matrix used to assess the feasibility of producing ASDs *via* different technologies. KSD is a versatile technology that is able to process APIs having diverse physicochemical properties.

## RECENT INNOVATIONS IN KinetiSol® DISPERSING

### Processing High Melt and High Viscosity Polymers—an Example with Polyvinyl Alcohol

As an example, PVA has known limitations on extrudability as its degradation temperature is very close to its melting temperature (154,155). Due to its semicrystalline nature, it will remain a solid below its melting point and is only extrudable at a temperature above its melting point (156). As the degradation temperature is close to the melting point, the HME process can easily cause degradation of the polymer. Therefore, the use of PVA for the preparation of ASDs, either using the HME process or spray drying, is very limited as it is thermally sensitive and has limited organic solubility. Brough et al. carried out a study to show how KSD can be used to prepare PVA-based ASDs with the model API itraconazole, using PVA as a solubility enhancing polymer for the poorly-soluble API (157). The study showed that KSD could effectively produce ASDs, and the optimal concentration of hydrolyzed grade PVA was 88%. Another study by LaFountaine et al. also demonstrated the feasibility of processing PVA as a polymer matrix to produce ASDs with ritonavir as the active ingredient using the KSD technology, even though ritonavir is shear sensitive and can be challenging to be processed (150). The study investigated the effect of KSD rotating speed and the ejection temperature on the physicochemical properties of the products to determine the optimal range of processing parameters for complete amorphous conversion with minimal to no API and polymer degradation. The study has proven that ASDs can be achieved with the KSD technology at a low-temperature range, from 80°C to 100°C, which is more than 60°C to 90°C below the degradation temperature of ritonavir and PVA respectively.

### Homogeneity with Very Low API Loading

Jermain et al. successfully produced homogenous ASDs of a high melting point, BCS class II drug, meloxicam, even at a very low API concentration of 1% *w*/w and with a short processing time of < 40 s (158). This is an important finding, showing that KSD technology can produce homogenous ASDs regardless of drug loading, which makes it suitable for formulating high potency APIs. Keen et al. reported an ASD system of itraconazole with high molecular weight HPMC using the KSD technology, in which the ASDs were then formed into tablets (159). The tablets containing the ASD showed a supersaturated concentration of itraconazole at basic pH. The dosage form containing the viscous solid dispersions of itraconazole, prolonged the absorption phase when being compared to the commercially available itraconazole tablets (Onmel®) (159).

### Improving upon a Commercialized ASD

Ellenberger et al. demonstrated the ability of KSD to produce an ASD of a poorly-soluble, low permeability API, vemurafenib (160). Producing vemurafenib ASDs has been challenging, and a method known as the solvent-controlled coprecipitation was developed to produce ASDs containing this API. The product is known as microprecipitated bulk powder (MBP) and is used commercially for Zelboraf® tablets produced by Roche (161). In this study, the vemurafenib ASD produced *via* KSD showed acceptable chemical purity and stability. It also has superior physicochemical properties when being compared with MBP. The morphology of the ASD produced by KSD was dense, smooth, and uniform, whereas the MBP was porous, resulting in a higher surface area. The KSD vemurafenib ASD has better dissolution and pharmacokinetic performance due to slower drug nucleation, recrystallization, and precipitation than the commercial formulation (160). Another study by Ellenberger et al. demonstrated an attempt of using KSD to produce a bioequivalent ASD of ritonavir that is double the drug load of a commercially available form of ritonavir (Norvir®), in which the composition can be made into a tablet dosage form with a mass of around 45% less than Norvir®(162). The KSD produced ritonavir tablets exhibited similar performance characteristics, such as the permeation rate and *in vivo* pharmacokinetics, to those of Norvir®. Lastly, Gala et al. demonstrated, for the first time, the ability of KSD to process short-chain cyclic oligomers with low molecular weights (*e.g.*, Hydroxypropyl-β-cyclodextrin [HPBCD]) (163). In this study, improved formulations were made of the generic abiraterone acetate tablets by creating ternary KSD products. Initially, binary HPBCD formulation with abiraterone experienced rapid dissolution in 0.01 N HCL but showed a poor ability to maintain drug supersaturation (*e.g.*, sometimes referred to as the parachute) effect in FaSSIF media. This lead to the eventual incorporation of the pH-dependent polymer HPMCAS 126 G, acting as a parachute to prevent abiraterone precipitation when transitioning to FaSSIF. The ternary ASD formulation selected (*e.g.*, HPBCD/HPMCAS 126 G/Abiraterone [80/10/10]) for *in vivo* studies in male beagle dogs achieved a 13.8-fold increase in bioavailability compared to the commercially available generic product (163).

### Amorphous Solid Dispersions Made by KSD Outperform Spray Dried Amorphous Solid Dispersions

**Fig. 10:**
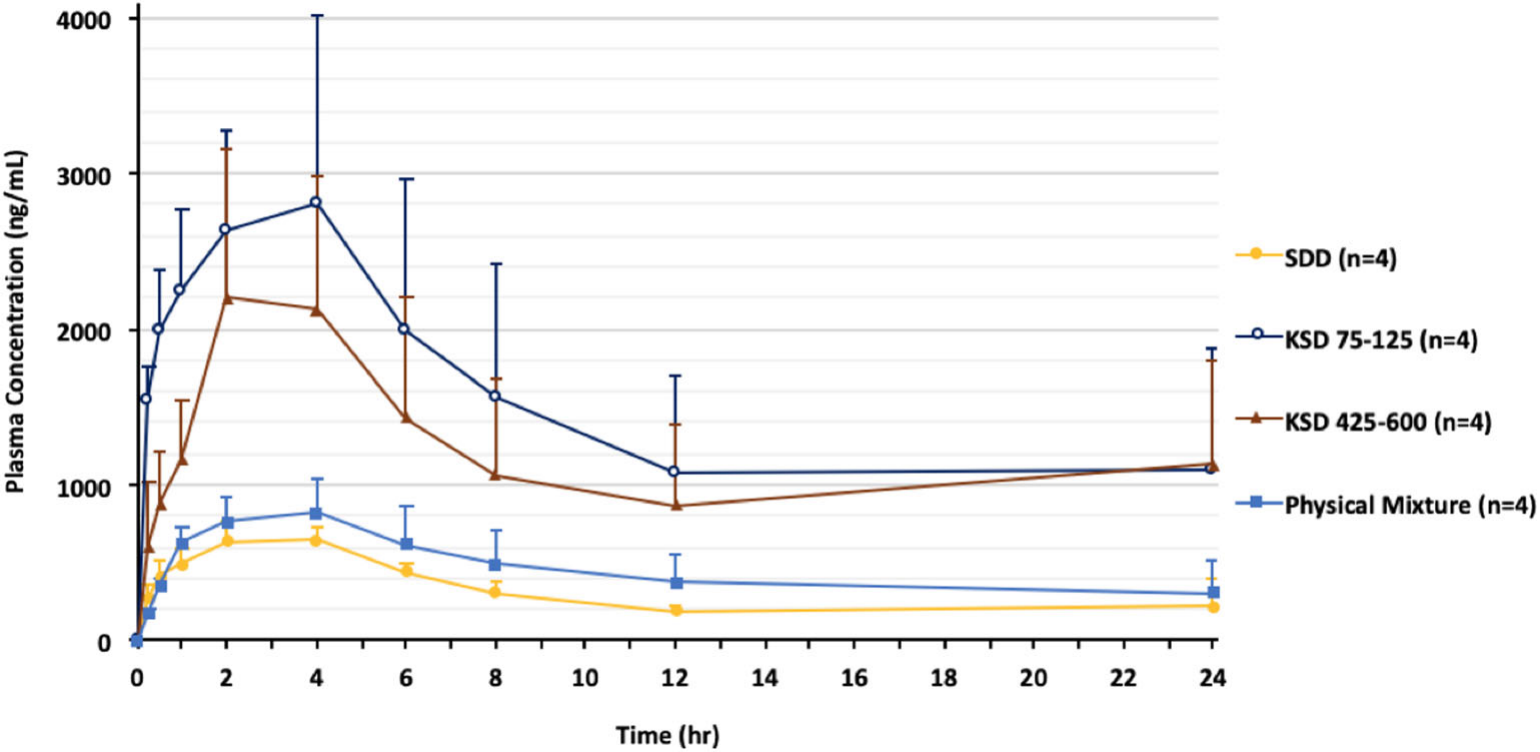
API plasma concentrations in male beagle dogs after oral administration of a 12 mg/mL suspension (4 mg equivalents of API/mL) at a target dose of 20 mg/kg. KSD refers to the Kinetisol ASD using that specific particle size. SDD refers to the spray-dried ASD. Adapted with permission from (164)

In a newly published study of a weakly basic API and ionic polymer, Jermain et al. report for the first time the effect of particle morphology that results from the manufacturing process on the rate and extent of drug release in both acidic and neutral media between ASDs manufactured by two different processing methods (*i.e.*, KSD and spray dried (SD)). The study found that the spray-dried and KinetiSol processed ASDs were equivalent at the molecular level; both samples demonstrate a high degree of miscibility and identical interactions when evaluated by solid-state NMR and FTIR. Despite both processes producing molecularly equivalent ASDs, significant differences were observed evaluating *in vivo* results in beagle dogs. The authors report the SD formulation achieved a similar bioavailability to the physical mixture; whereas, the KSD formulation experienced a 3-fold increase in bioavailability (Fig. 10). The performance differences arise from the KSD process producing dense particles with decreased specific surface area (158,160,164), in comparison to the spray-dried process that generates particles with high porosity and high specific surface area (164,165). The higher specific surface area increased the probability for the API to be exposed on the particle surface and not be protected by the polymer, explaining the increased dissolution exhibited by the spray-dried ASD formulation. In the case with HPMCAS-HMP, the polymer is insoluble in the unionized state below pH 6; therefore, API experiencing supersaturation in acidic conditions cannot be stabilized by the polymer and thus quickly recrystallize upon transition to neutral conditions. The dense particles produced by the Kinetisol process minimized the amount of weakly basic drug-exposed on the surface of the particle; this ultimately minimized drug release in acidic conditions thus protecting the API from recrystallization and allowed the formulation to experience supersaturation in the small intestine where the molecule is absorbed (164). In summary, rapid dissolution of the SD particles in the acidic phase, attributed to their high specific surface area, rapidly recrystallized during the pH transition as the enteric polymer was not able to stabilize the API; whereas, the dense KSD particles lower surface area protected the API, allowing for drug and polymer to release congruently after the pH-shift, preserving the amorphous nature and increasing bioavailability. Though not evaluated in this study, it would be expected from the data on dissolution performance that the spray-dried ASD would have a higher tendency to recrystallize during storage from the increased exposure of the API on the surface to be in contact with moisture. This study establishes KinetiSol as a superior formulation process compared to spray drying for developing ASDs of weakly basic APIs and ionic polymers.

### Expanding the Formulation Design Space of ASDs

**Fig. 11:**
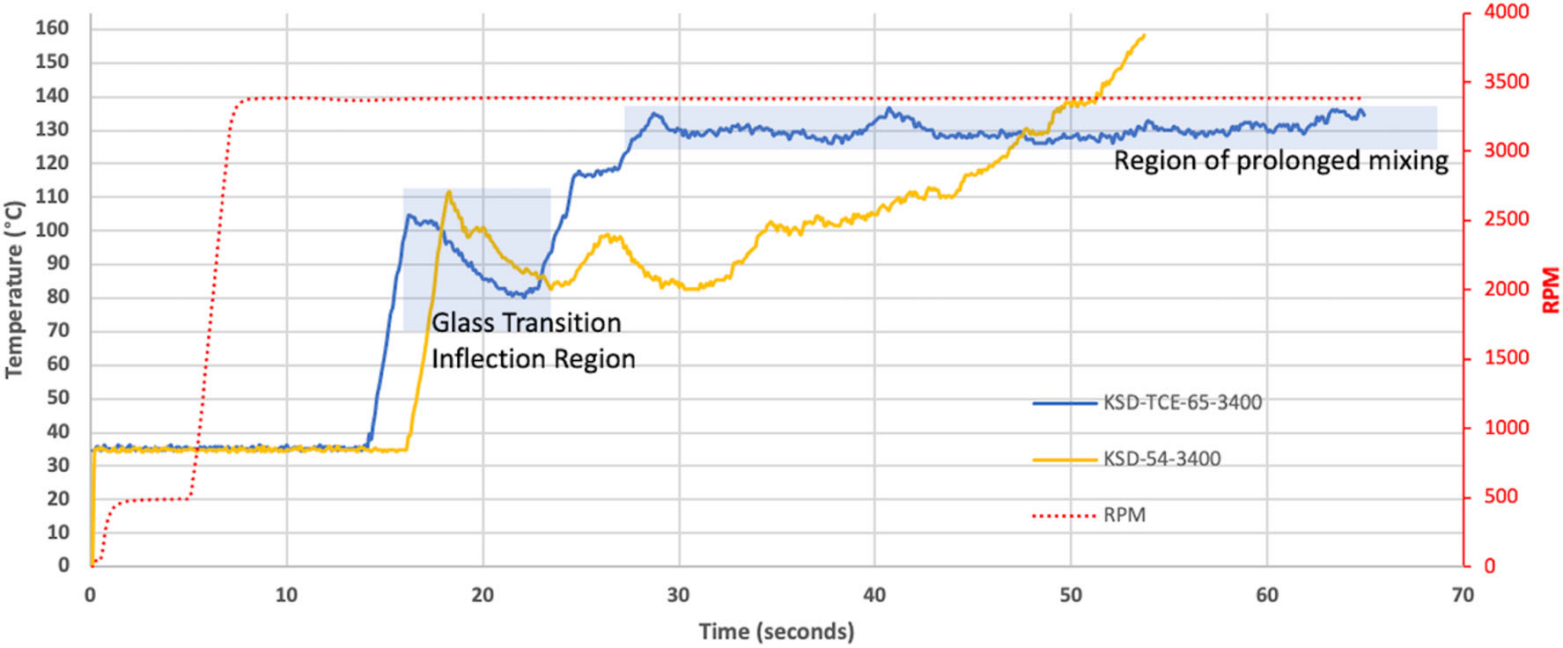
This figure compares the outcomes on the processing profiles when a KSD and KSD-TCE composition are exposed to the same processing conditions. Adapted with permission from (166)

As discussed, thermal processes rely on drug dissolution into a polymer matrix to form ASDs when processed below the APIs melting point (157–160,164). KinetiSol’s high-energy mixing conditions mean that it can sufficiently mix and render an API amorphous in less than 5 s at elevated temperatures (150). The rapid increase in the composition’s temperature during the process results from the mechanical energy applied to the system in combination with the inability of the composition to dissipate the heat generated from the process (166). In a recent study that has expanded the design space of the KinetiSol process, Davis et al. demonstrated the advantage of improving the thermal conductivity of a composition within the KinetiSol process. The addition of a thermally conductive excipient, candurin, allowed the composition to dissipate the energy efficiently, creating a steady-state processing condition where the composition experienced a region of prolonged mixing at a constant and controlled temperature (Fig. 11). The precise control over the processing conditions eliminates unnecessary exposure to elevated temperature as well, ensuring homogenous mixing at lower processing temperatures. In addition to increasing the mixing period at lower temperatures, a decreased processing temperature can be utilized with APIs that are incredibly heat sensitive or shear sensitive; these less aggressive processing conditions could be favorable in eliminating API degradation to these sensitive APIs.

### Process Analytical Tools for KinetiSol® Dispersing

Currently, an infrared probe is the standard PAT in all KSD processes to monitor the temperature of the materials in the chamber real-time and rapidly eject once the desired temperature is achieved (164). The KSD chamber can be equipped with additional probe ports in order to simultaneously monitor multiple streams of spectroscopic data during Kinetisol® dispersing;. DisperSol is currently developing control schemes employing various monitoring tools. This potentially enables ejection based on temperature, degree of processing, and/or crystallinity. The ability to carry out a prediction in real-time makes KSD an appealing approach for preclinical product development as well as commercial GMP manufacturing.

## CONCLUSION

The HME and KSD technologies are capable of converting a poorly water-soluble, crystalline API into an amorphous state by dispersing it into a polymeric matrix, effectively forming an amorphous solid dispersion. When producing ASDs *via* HME, the melting point of the API often needs to be compatible with the melting point of the polymer for the successful conversion of a stable ASD. The melting point incompatibility may cause degradation and instability issues. The introduction of plasticizers during the HME process seems to overcome the high processing temperature issue, but problems with instability may be propagated. HME can also be utilized as a reaction vessel to form co-crystals, another viable way to improve the bioavailability of poorly water-soluble APIs. One of the barriers to using HME for co-crystal production is overcoming the tendency of co-crystals to form hydrates in the presence of water, causing instability. To overcome this issue, a novel co-crystal formulation, known as MAC, was developed by introducing a polymeric matrix to improve its physicochemical properties. The HME technology can be further extended by incorporating commonly used characterization and monitoring tools such as FT-NIR, Raman, and UV-Vis spectroscopies to achieve real-time monitoring of the product during the HME process. The capability of in-line monitoring allows effective product development and allows a deeper understanding of the influence of processing parameters on the quality of the products. KSD technology was developed as a process to formulate high melting point APIs with any polymeric carrier to form a stable ASD. One interesting feature of the KSD is that there is no external heat applied during the process; moreover, the heat was solely generated through the friction and shearing of the materials and the blades during the process. The KSD process imparts very high energy due to high rotating speeds of more than 1000 rpm, allowing the formation of stable ASDs with short processing times and lower processing temperatures. KSD and spray-drying technologies produced molecularly similar ASDs, but due to differences in specific surface area and porosity, the KSD ASD had superior bioavailability in *in vivo* studies. Lastly, the addition of thermally conductive excipients to polymeric compositions has increased the KSD design space, allowing processing for prolonged periods at fixed-controllable temperatures. There are now rapid innovations in both HME and KSD processes to further expand their utility in improving solubility and bioavailability of poorly water-soluble APIs.
